# Efficacy of chemo-mechanical caries removal: a 24-month randomized trial

**DOI:** 10.3389/froh.2024.1458530

**Published:** 2024-12-03

**Authors:** Kemporn Kitsahawong, Ana Lucia Seminario, Patimaporn Pungchanchaikul, Anoma Rattanacharoenthum, Pipop Sutthiprapaporn, Waranuch Pitiphat

**Affiliations:** ^1^ Department of Preventive Dentistry, Faculty of Dentistry, Khon Kaen University, Khon Kaen, Thailand; ^2^ School of Dentistry, Timothy A. DeRouen Center for Global Oral Health, University of Washington, Seattle, WA, United States; ^3^ Department of Pediatric Dentistry, School of Dentistry, Universidad Peruana Cayetano Heredia, Lima, Peru

**Keywords:** dental caries, dental atraumatic restorative treatment, papain, glass ionomer cements, deciduous tooth

## Abstract

**Introduction:**

Chemo-mechanical caries removal (CMCR) offers an alternative to conventional drilling for carious dentin removal, but limited evidence exists on its long-term effects on treating caries in primary teeth. The primary aims of this study were to compare CMCR to drilling in terms of restoration status and the presence of secondary caries after 24 months.

**Methods:**

A randomized, parallel-group, single-blinded, controlled trial was conducted in Thailand. Participants were children aged 7–8 years with occlusal caries in second primary molars, randomly assigned to CMCR with Papacarie® (*n* = 242) or drilling (*n* = 246). All cavities were restored using glass ionomer cement. Completeness of caries removal was evaluated clinically by two blinded dentists, and patients' discomfort was measured using a facial visual analogue scale. Restoration status and development of secondary caries were assessed every six months clinically and every 12 months radiographically over a 2-year period.

**Results:**

Both groups achieved complete caries removal, but the CMCR took significantly longer than the drilling method (9 vs. 2.3 min, *p* < 0.001). Children in the CMCR group reported significantly lower discomfort during treatment (*p* < 0.001). At 24 months, ten (4.6%) restorations in the CMCR group failed, compared to three (1.2%) in the drilling group. Clinically, four restorations (CMCR = 2, control = 2) exhibited secondary caries at the margin, while nine restorations (CMCR = 8, control = 1) showed radiographic evidence of secondary caries beneath the restoration. However, neither clinical nor radiographic evaluations revealed statistically significant differences in treatment outcomes at the two-year mark.

**Conclusion:**

CMCR demonstrated comparable efficacy to conventional drilling for complete caries removal and restoration success at 24 months in primary teeth. Despite a longer chair time, it resulted in less discomfort during treatment.

**Clinical Trial Registration:**

https://clinicaltrials.gov/study/NCT01641861, identifier: NCT01641861.

## 1 Introduction

Growing evidence and advancements in cariology increasingly support a paradigm shift towards minimal intervention techniques in dental caries treatment (1). This shift is particularly relevant during the COVID-19 pandemic, where chemo-mechanical caries removal (CMCR) has emerged as a valuable alternative to conventional methods due to its reduced aerosol generation (2). In minimal intervention dentistry, the goal is to selectively remove only the infected carious dentine while leaving sound tooth structure intact. Conventional caries removal with rotary metal burs, however, may inadvertently remove sound dentine. Moreover, the heat and vibration generated during drilling can adversely impact pulp tissue vitality, potentially causing discomfort and pain in patients and necessitating local anesthesia (3–6). These challenges, especially in managing caries in children's primary teeth, highlight the potential of CMCR.

One promising CMCR technique utilizes Papacarie®, a gel introduced in 2003 containing papain, a plant enzyme with bacteriostatic and anti-inflammatory properties. This enzyme selectively digests degraded tissues, allowing for the targeted removal of infected dentine using spoon excavators (7–9). Unlike damaged infected dentine, affected dentine can be remineralized, making it advisable to leave it *in situ*. Subsequently, the prepared cavity can be restored with materials like glass ionomer cement (GIC), which chemically bonds to the tooth structures and promotes remineralization.

A critical review of global research on CMCR products found few studies evaluating restoration survival in primary teeth compared to conventional methods (2). We have previously demonstrated *in vitro* that there was no significant difference in the completeness of caries removal between Papacarie® and the traditional drilling technique. This assessment was made using visual and tactile criteria as well as a caries detector device (10). While limited published clinical reports exist on Papacarie®, they suggest it offers a less traumatic approach to caries removal with a lower incidence of pulpal exposure than traditional drilling with rotary burs (11). To address this gap, we conducted a randomized controlled trial with the primary aims to evaluate the clinical efficacy of Papacarie® vs. the conventional drilling method concerning restoration status and the development of secondary caries after 24 months. Additionally, we compared the efficacy of caries removal, treatment duration, and levels of pain and discomfort associated with the two methods.

## 2 Materials and methods

### 2.1 Study design

This study was a randomized, parallel-group, single-blinded, controlled clinical trial. The protocol received approval from the institutional review boards of Khon Kaen University (approval number: HE542161) and the University of Washington (approval number: 41189). The trial was registered prior to participant enrollment at the ClinicalTrials.gov Protocol Registration and Results System (PRS) (NCT01641861). It was conducted in accordance with the principles of the Declaration of Helsinki and reported following the Consolidated Standards of Reporting Trials (CONSORT) Statement (12). Informed consent was obtained from the parents or legal guardians for the child to participate in the study.

### 2.2 Participants

We recruited children aged between 7 and 8 years with at least one active caries lesion in a second primary molar. The recruitment took place among students attending primary schools in Khon Kaen, Thailand, from June 2012 to August 2015. Those children with signed consent forms were pre-screened at school by an investigator (K.K.). Individuals who appeared eligible were invited to the Pediatric Dentistry Clinic at Khon Kaen University Faculty of Dentistry for clinical and radiographic examinations to confirm their eligibility. Children were not enrolled if they were uncooperative, were medically classified as ASA III or more (American Society of Anesthesiologists) or exhibited known allergic reactions to ingredients contained in the materials used. To be included in the trial, children's teeth had to be vital, with occlusal caries affecting no more than two-thirds of the dentine layer evaluated by radiographic examinations, and without spontaneous pain. Additionally, the carious cavities had to be large enough to be operated on with hand instruments. Teeth were excluded if they exhibited pathological conditions other than caries, as determined by clinical and radiographic examinations. If children had more than one eligible carious tooth, only one tooth was randomly selected to receive the assigned intervention.

### 2.3 Randomization

Willing and eligible participants were randomly allocated to the intervention and control groups with a 1:1 allocation ratio. The random allocation sequence was generated by a researcher (W.P.) who was not involved in the examination, using a random number-producing algorithm (Research Randomizer V4.0, http://www.randomizer.org/form.htm). Concealed envelopes containing the generated codes were kept in containers until interventions were assigned by a dental assistant.

### 2.4 Treatment procedures

The intervention involved CMCR using Papacarie®, while the control group underwent the conventional drilling method. We followed the manufacturer's instructions for Papacarie® administration (30–60 s) to the dental cavity to soften the carious dentine. Subsequently, dental caries was gently removed using hand instruments. The procedure was repeated until the gel no longer exhibited clouding and the lesion surfaces felt hard. We used GC dentine conditioner (a 10% polyacrylic acid solution) to clean the tooth surfaces before restoring them with GIC filling material (GC Fuji IX GP EXTRA, GC corporation, Tokyo, Japan) (Supplementary Figure S1).

For participants in the control group, dental caries was removed using the conventional drilling method. Briefly, the process involved the use of an airotor with a carbide bur number 330, followed by a low-speed air motor with a round steel bur. Restorations were carried out in the same way as in the CMCR group (Supplementary Figure S2).

All treatments were performed by one calibrated pediatric dentist (K.K.). The restorative procedures were conducted using rubber dam isolation with Dental Dam Stabilizing Cord (Wedjets, Coltène/Whaledent Inc., Cuyahoga Falls, OH, USA). No local anesthesia injection was administered in this study, as no participant requested it.

### 2.5 Outcome measurements

The primary outcomes included restoration status (failure/success) and the presence of secondary caries after 24 months. The secondary outcomes, measured during the treatment visit, comprised the efficacy of caries removal, treatment duration, and levels of pain and discomfort.

#### 2.5.1 Evaluations during the treatment visit

The time taken for caries removal was recorded, starting from the application of gel or the beginning of drilling until the completion of caries removal procedures. The time for treatment encompassed the period from applying the gel or starting drilling to the completion of the restoration.

The completeness of caries removal was clinically judged based on visual and tactile criteria: no discoloration visually and smooth explorer passage with no catch or “tug-back” sensation tactually (11, 13). Two blinded examiners (A.R., P.P.) underwent training and calibration in a laboratory setting before examining the participants. Both examiners demonstrated excellent intra-examiner agreement, with one achieving 100% and the other achieving 95%. The inter-examiner agreement was also high, at 95%.

Patient perception of pain and discomfort was evaluated using the facial Visual Analogue Scale (VAS) and recorded twice: once before treatment and once after the completion of caries removal. The VAS instrument has two sides: one side displays a 100-millimeter ruler scale, while the other side is a VAS composed of six facial expressions. The happiest face represents smiling, the saddest face represents crying, and the intermediate faces depict varying degrees of happiness and sadness. The VAS instrument was given to the patient with the instruction, “If you were this face right now, which one would you be?” The patient would then point to the corresponding face that best represented their degree of pain or discomfort. The score was recorded on the ruler scale (0 to 100 millimeters, with 0 indicating no pain and 100 indicating extreme pain).

#### 2.5.2 Follow-up evaluations

The restoration status and development of secondary caries were clinically assessed every six months and radiographically assessed every 12 months over a 2-year period. Two blinded examiners (A.R., P.P.), who were not involved in enrollment, randomization, or treatment, conducted the clinical evaluations using criteria modified from the United States Public Health Service (USPHS) guidelines (14). Assessments included six parameters: retention, color match, cavosurface marginal discoloration, anatomic form, marginal adaptation, and secondary caries. The restoration status was then determined as either success or failure, as described in Table 1.

**Table 1:** Clinical evaluation of restoration status using modified United States public health service (USPHS) criteria.

Category	Restoration status		Criteria
	Success	Failure	
*Retention*	Alpha		Restoration is present.
		Bravo	Restoration is partially or totally missing.
*Color match*	Alpha		Restoration matches adjacent tooth structure in color, shade, or translucency.
	Bravo		There is a mismatch in color, shade, or translucency but within the normal range of adjacent tooth structure.
		Charlie	There is a mismatch in color, shade, or translucency outside of the normal range of adjacent tooth structure.
*Cavosurface marginal discoloration*	Alpha		There is no discoloration anywhere on the margin between the restoration and the tooth structure.
	Bravo		Discoloration is present but has not penetrated along the margin in a pulpal direction.
		Charlie	Discoloration has penetrated along the margin in a pulpal direction.
*Anatomic form*	Alpha		The restoration is continuous with existing anatomic form.
	Bravo		The restoration is discontinuous with existing anatomic form, but missing materials are not sufficient to expose dentin or base.
		Charlie	Sufficient restorative material is missing expose the dentin or base.
*Marginal adaptation*	Alpha		There is no visible evidence of a crevice along the margin into which the explorer will penetrate.
	Bravo		There is visible evidence of a crevice along the margin into which the explorer will penetrate or catch.
		Charlie	The explorer penetrates the crevice, and dentin or base is exposed.
		Delta	The restoration is mobile, fractured, or missing, either in part or total.
*Secondary caries*	Alpha		No caries is present at the margin of the restoration, as evidenced by softness, opacity, or etching at the margin.
		Bravo	There is evidence of caries at the margin of restoration.

Bitewing radiographs were taken using conventional No. 0 intraoral film in conjunction with the XCP dental x-ray film holder as positioning and guidance. These radiographs were independently examined by an oral and maxillofacial radiologist (P.S.) with 15 years of experience, who was unaware of participants' allocation, at 12 and 24 months after treatment, following the criteria used by Munshi et al. (15). Absence of restorations and/or signs of caries on the radiographs were classified as failures. All radiographs underwent re-evaluation after a minimum of one month, and there was 100% agreement on the classification of success/failure in the two examinations.

Failure of any parameter in clinical or radiographic assessments was considered a restoration failure. Restorations with failing assessments underwent re-treatment, which was excluded from further evaluations.

### 2.6 Sample size

We calculated the sample size for each primary aim, choosing the largest sample number to address all study questions. Prior data indicated a 25% probability of unacceptable restoration in the conventional group and 14% in the chemo-mechanical group (16). Based on a power of 80% and a significance level of 5%, we needed to enroll 203 participants in each group to be able to reject the null hypothesis that the failure rates of the two comparison groups are equal. Considering a 20% loss to follow-up, we increased the number of participants to 244 in each group.

### 2.7 Statistical analysis

We used the Shapiro-Wilk test to assess the normality of continuous variables. We employed the Mann-Whitney *U*-test to compare group differences for skewed continuous variables, and chi-square test to analyze categorical variables. Logistic regression analysis was employed to analyze the failure rate of treatment, based on the clinical and radiographic evaluations of restoration status at 6, 12, 18, and 24 months. We analyzed the data using both intention-to-treat (ITT) and per-protocol (PP) approaches. In the ITT analysis, missing data were imputed using the last observation carried forward method in which the last available data at the time point before participants lost from the study were retained in the analysis. In comparison, the PP analysis included only the subjects who remained in the study at the 24-month follow-up visit. The analyses were performed using STATA version 10 (StataCorp, College Station, Texas, USA). All statistical tests were two-sided with a significance level of 5%.

## 3 Results

### 3.1 Characteristic of participants

**Figure 1:**
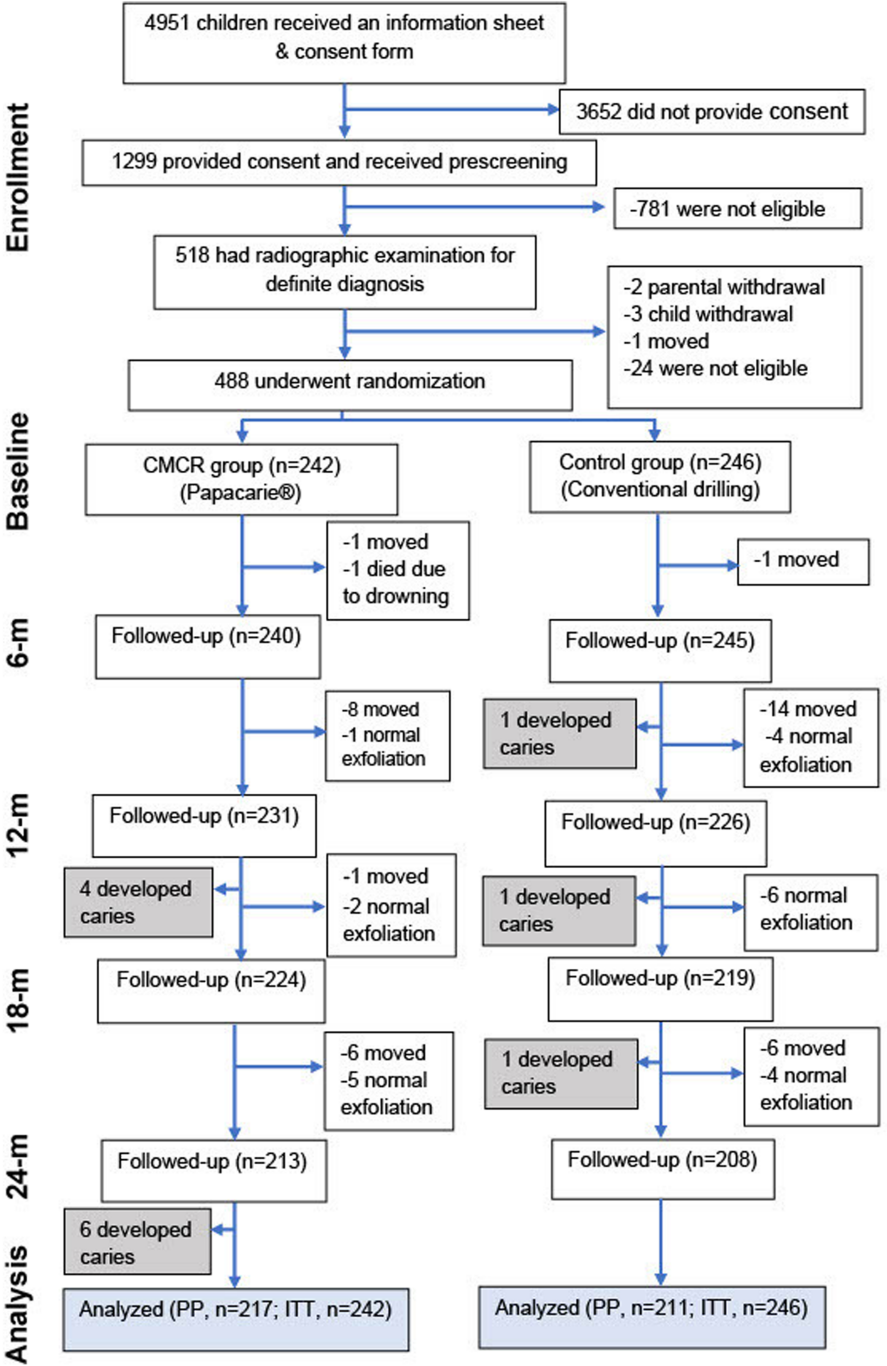
Flow diagram of participant enrollment, randomization, follow-up, and analysis. CMCR, chemo-mechanical caries removal; PP, per-protocol; ITT, intention-to-treat.

Figure 1 displays the participant flow diagram. Of the 488 enrolled children, 242 were randomized into the CMCR group, while another 246 children were allocated into the control group. Children in the CMCR group had a significantly higher number of carious primary teeth at baseline compared to those in the control group (*p* < 0.001). There were no differences between the two groups in terms of sex, age, total number of teeth, number of permanent teeth with caries experience, and the presence of malocclusion. Teeth selected for treatment in the CMCR group were more frequently located in the upper jaw and had deeper caries lesions compared to controls (Table 2).

**Table 2:** Baseline characteristics of study participants by treatment group.

	CMCR (Papacaries®) (*n* = 242)	Control (Drilling) (*n* = 246)	*p*-value
Participant characteristics			
Male, *n* (%)	126 (52.1)	117 (47.6)	0.32^a^
Age in months			
Mean (SD)	89.2 (7.5)	89.9 (8.4)	
Median (IQR)	88 (84–96)	89 (84–96)	0.49^b^
Number of primary teeth			
Mean (SD)	15.2 (2.8)	15.1 (3.0)	
Median (IQR)	15 (13–18)	15 (12–18)	0.69^b^
dmft			
Mean (SD)	8.6 (3.2)	7.7 (3.5)	
Median (IQR)	9 (6–10)	7 (5–10)	0.001^b^^,^*
Number of permanent teeth	(*n* = 236)	(*n* = 237)	
Mean (SD)	7.9 (3.3)	8.0 (3.6)	
Median (IQR)	8 (6–10)	8 (6–11)	0.69^b^
DMFT	(*n* = 236)	(*n* = 237)	
Mean (SD)	1.0 (1.2)	0.9 (1.2)	
Median (IQR)	0 (0–2)	0 (0–2)	0.18^b^
Malocclusion, *n* (%)	1 (0.4)	0 (0)	0.31^a^
Having sealant, *n* (%)	67 (27.7)	57 (23.2)	0.25^a^
Urgent treatment need, *n* (%)	6 (2.5)	4 (1.6)	0.50^a^
Treated tooth characteristics			
Dentition, *n* (%)			0.01^a^^,^*
Upper	146 (60.3)	120 (48.8)	
Lower	96 (39.7)	126 (51.2)	
Size of caries, *n* (%)			0.10^a^
Small	1 (0.4)	0 (0)	
Medium	208 (86.0)	225 (91.5)	
Large	25 (10.3)	19 (7.7)	
Extra-large	8 (3.3)	2 (0.8)	
Depth of caries, *n* (%)			0.001^a^^,^*
1/3 of dentine	45 (18.6)	120 (48.8)	
1/2 of dentine	125 (51.7)	108 (43.9)	
2/3 of dentine	72 (29.8)	18 (7.3)	

CMCR, chemo-mechanical caries removal; SD, standard deviation; IQR, interquartile range.

^a^ Chi-square test.

^b^ Mann-Whitney *U*-test.

* statistically significant at *p* < 0.05.

### 3.2 Efficacy of caries removal, treatment duration, and patient perception

No residual caries remained after caries removal using both methods. However, the CMCR group required significantly more time for both caries removal (*p* = 0.001) and the total treatment time (*p* = 0.001) compared to the control group (Table 3). The VAS scores for pain and discomfort in the CMCR group were significantly higher before treatment than in the control group (*p* = 0.004) but were significantly lower during treatment (*p* = 0.001). No participant in either group requested local anesthesia either before or during the procedure.

**Table 3:** Procedure duration and patient perception of pain and discomfort by treatment group.

Outcome measure	CMCR® (Papacarie) (*n* = 242)	Control (Drilling) (*n* = 246)	*p*-value^a^
Time use for caries removal (minutes)			
Mean (SD)	9.0 (2.9)	2.3 (1.5)	
Median (IQR)	8.5 (6.7–10.6)	1.7 (1.5–2.5)	0.001
Time use for treatment (minutes)			
Mean (SD)	14.1 (3.2)	7.5 (1.9)	
Median (IQR)	13.9 (11.8–16.5)	7.1 (6.6–7.9)	0.001
Pain and discomfort score: before treatment			
Mean (SD)	24.3 (18.9)	19.6 (15.7)	
Median (IQR)	23 (7–40)	12 (7–27)	0.004
Pain and discomfort score: after treatment			
Mean (SD)	32.9 (17.9)	47.9 (19.9)	
Median (IQR)	32 (22–43)	45 (33–60)	0.001

CMCR, chemo-mechanical caries removal; SD, standard deviation; IQR, interquartile range.

^a^ Mann-Whitney *U*-test.

### 3.3 Restoration status and presence of secondary caries after 2 years

Out of 488 children, 428 (87.7%) contributed the information for the PP analysis at the 24-month follow-up (shown in Figure 1). Reasons for dropouts included participant's relocation (*n* = 37), natural exfoliation of the treated teeth (*n* = 22), and death due to drowning (*n* = 1). Two examiners separately evaluated the outcomes. At each follow-up visit, at least a 10% random sample of patients was re-evaluated by a different examiner to determine the reliability of the assessments. The excellent intra-examiner agreements for clinical findings ranged from 95% to 97%, and the inter-examiner agreement was also high, at 93%.

At 24 months, ten (4.6%) restorations in the CMCR group failed, compared to three (1.4%) in the control group (Table 4). All failures were due to secondary caries. Of these failures, four restorations (CMCR = 2, control = 2) exhibited secondary caries at the margin as observed clinically, while nine (CMCR = 8, control = 1) showed evidence of secondary caries beneath the restoration on radiographic examination.

**Table 4:** Failure rate (%) by treatment group. .

Follow-up visit	Intention-to-treat analysis			Per-protocol analysis		
	CMCR (Papacarie®)	Control (Drilling)	Odds Ratio (95% CI)	CMCR (Papacarie®)	Control (Drilling)	Odds Ratio (95% CI)
6-month	0/242 (0)	1/246 (0.4)	10.3 (0.01–8.3)	0/240 (0)	1/245 (0.4)	10.3 (0.01–8.4)
12-month	4/242 (1.7)	2/246 (0.8)	2.1 (0.4–11.3)	4/231 (1.7)	2/227 (0.9)	2.0 (0.4–10.9)
18-month	4/242 (1.7)	3/246 (1.2)	1.4 (0.3–6.1)	4/228 (1.8)	3/221 (1.4)	1.3 (0.3–5.9)
24-month	10/242 (4.1)	3/246 (1.2)	3.5 (0.9–12.8)	10/217 (4.6)	3/211 (1.4)	3.3 (0.9–12.3)

CMCR, chemo-mechanical caries removal; CI, confidence interval.

.

Logistic regression analysis showed that restorations in the intervention group were approximately three times as likely to fail compared to those in the control group, but the results were not statistically significant for both ITT and PP analyses (ITT: OR 3.5, 95% CI 0.9–12.8; PP: OR 3.3, 95% CI 0.9–12.3). Additional adjustment for characteristics which were imbalanced between the two groups, including tooth location, depth of caries, and dmft, did not change the results substantially (ITT: OR 3.6, 95% CI 0.9–14.3; PP: OR 3.5, 95% CI 0.9–14.0).

## 4 Discussion

Caries removal is a critical procedure for determining successful clinical outcomes, partly by ensuring the integrity of cavity walls and preventing recurrent/secondary caries. In this study, we examined the efficacy of caries treatments both clinically and radiographically, focusing on restoration status and secondary caries development at 24 months after treatment. Our findings support the hypothesis that CMCR could be as effective as the conventional drilling method. Specifically, we found that no residual caries remained after caries removal using both methods. The VAS scores for pain and discomfort were significantly lower after treatment in the CMCR group compared to the control group. Additionally, the difference in restoration failures between the CMCR group and the control group was not statistically significant.

Marginal integrity of the cavity strongly influences the quality of the cavity seal and the potential for microleakage (17). Irregularity of the cavity wall resulting from excavation and reduced surface hardness of the affected dentine could make it less suitable for the adaptation of restorative materials, potentially contributing to the observed marginal leakage (10, 18). The present study observed secondary caries in both groups, either at the cavity margin (*n* = 4) or radiographically detected underneath restorations (*n* = 9), after 24 months. However, there was no statistically significant difference in the incidence of secondary caries between the comparison groups.

The present study was conducted on the primary molars using high viscosity GIC as a restorative material. This material was chosen for its good clinical performance, low technical sensitivity, and simplicity in manipulation with young, uncooperative patients, thereby eliminating the need for rubber dam placement (19). Despite the potentially compromised marginal integrity, the treatment success rate in the Papacarie® group remained high at 96%. This may be attributable to the properties of GIC, which compensate for the disadvantages of the CMCR method. Anticipated benefits of GIC include the ability to initiate remineralization of affected dentine and acting as a reservoir for continuous fluoride release to reduce the risk of future caries (20–22). Theoretically, the ionic bonding of GIC to dental tissue gradually matures to create a seal that could block bacteria re-entry from plaque. The seal also provides a perfect environment for the remineralization of demineralized dentine walls (23). Furthermore, Papacarie® effectively reduces residual cariogenic bacteria in carious lesions (24–26). A systematic review and meta-analysis concluded that Papacarie® left significantly fewer bacteria after caries removal compared to the conventional method (mean difference 0.57 log10 CFU, 95% CI 0.04–1.09, based on two studies) (27). Our findings align with a previous case series in primary teeth, where Papacarie® demonstrated high clinical (88%) and radiographic success rates (99%) at a 1-year follow-up (28).

Limited randomized controlled trials have compared the long-term outcomes of restoration using Papacaries® and conventional drilling for removing caries in primary molars. A small split-mouth trial involving 20 children reported a significantly higher success rate in the Papacaries® group compared to drilling with low-speed burs (95% vs. 80%) at the 18-month follow-up (29). Our study, however, found no statistically significant difference in the failure rate between the two comparison groups. Although treatment with Papacarie® was three times more likely to fail at the 24-month follow-up than in the control group, the result did not reach statistical significance (unadjusted OR 3.3, 95%CI 0.9–12.3). Based on our findings, both CMCR and the conventional drilling method appear to be effective for treating caries in primary molars.

Caries removal using the CMCR method may not be suitable for small lesions due to limited access and visibility (30). However, even when selecting accessible lesions, the mean caries removal time with CMCR was significantly longer than with drilling in our study, consistent with most previous studies (6, 10, 11, 31, 32). In contrast, Matsumoto et al. (2013) and Motta et al. (2014) reported no significant difference in caries removal time between Papacarie® and drilling methods (21, 29). Our study found a mean excavation time of 2.3 min for drilling and 9.0 min for the Papacarie® method, aligning with a meta-analysis reporting 2.99 min for rotary caries removal, and 6.36 min for the CMCR method (33). Therefore, longer treatment times might be a drawback of Papacarie®. However, the avoidance of local anesthesia with Papacarie® can potentially reduce the overall procedure time by eliminating injection, waiting for anesthesia onset, and behavior management (34).

Dental anxiety is a common barrier for patients seeking oral health care. Pain and discomfort during drilling are frequently reported, and several methods, including CMCR (6, 35–38), sonic and ultrasonic devices (39), and lasers (40), have been suggested for cavity preparation to help alleviate this pain. The present study determined the degree of discomfort experienced during caries removal using the VAS evaluation with a face scale, which has been proven to be an effective tool for children (41). While no participant in this study requested local anesthesia, those in the intervention group experienced less pain and discomfort during treatment compared to those treated with conventional drilling. This could be attributed to the neutral pH of the gel and the local action of papain in the repair process (35), which is less likely to disturb pulp tissue. When compared to using drilling burs, the use of a blunt spoon excavator appeared to be gentler and less likely to accidentally expose the pulp, thereby reducing the likelihood of stimulating the child's sensation. Our results were similar to those of a previous study comparing the conventional drilling, Carisolv, and Papacarie® methods for caries removal in primary teeth. The study reported that using Papacarie® was the most preferred method for caries removal, while the rotary drilling method was the least preferred among children (42).

Based on our results in 7–8-year-old children, CMCR using Papacarie® followed by GIC restoration may be advantageous for restoring primary molars in younger-aged children. However, for patients with limited cooperation, such as those with intellectual disabilities, a more suitable behavior management technique would require shorter treatment times, possibly necessitating the use of drilling burs. We recommend the CMCR caries treatment technique for resource-constrained settings. The limitation of the CMCR method is that, if the cavity caries cannot be accessed by the instrument, it cannot be done. However, due to the limited cavity access of the CMCR method, additional use of rotary instruments may be necessary, for example, to remove undermined enamel in cases of occult caries or for class II cavity preparation.

Our study had limitations. Firstly, despite randomization, there were still imbalances in certain baseline characteristics between the two comparison groups, including the location and depth of the treated teeth. The depth of caries could significantly impact the treatment procedure and various outcomes, such as treatment time, participants' comfort, restoration status, and secondary caries development. In this study, teeth selected for treatment in the CMCR group exhibited deeper caries lesions compared to controls. Consequently, it is unlikely that this difference would explain the positive outcomes observed in the CMCR group. Secondly, the long duration of the trial required high compliance from patients, leading to some participant loss. Therefore, we anticipated attrition and ensured that the final number of participants in each group met the required sample size. Thirdly, neither the operator nor the patients were blinded to the intervention group due to the obvious differences between the intervention techniques. However, for clinical and radiographic evaluations of the primary outcomes—restoration status and secondary caries—examiner blinding could be achieved since the restorations in both groups were identical in material. Finally, in this study, a single trained operator performed all procedures to minimize inter-operator variability. Variations in the use of these techniques among different operators may affect the generalizability of the outcomes and warrant further investigation in future clinical studies.

## 5 Conclusion

This study found no significant difference between CMCR and the conventional drilling method in terms of caries removal efficacy, restoration failure and secondary caries after two years. While CMCR with Papacarie® required longer excavation time, it led to reduced pain and increased patient comfort compared to the conventional method. Therefore, the results demonstrate that Papacarie® is an effective alternative for caries removal in primary teeth.

## Data Availability

The raw data supporting the conclusions of this article will be made available by the authors, without undue reservation.
