# Supplementary figures and images for: Efficacy of chemo-mechanical caries removal: a 24-month randomized trial
Source: Front Oral Health. 2024 Dec 3;5:1458530. doi: 10.3389/froh.2024.1458530 (PMC11653365; doi:10.3389/froh.2024.1458530)

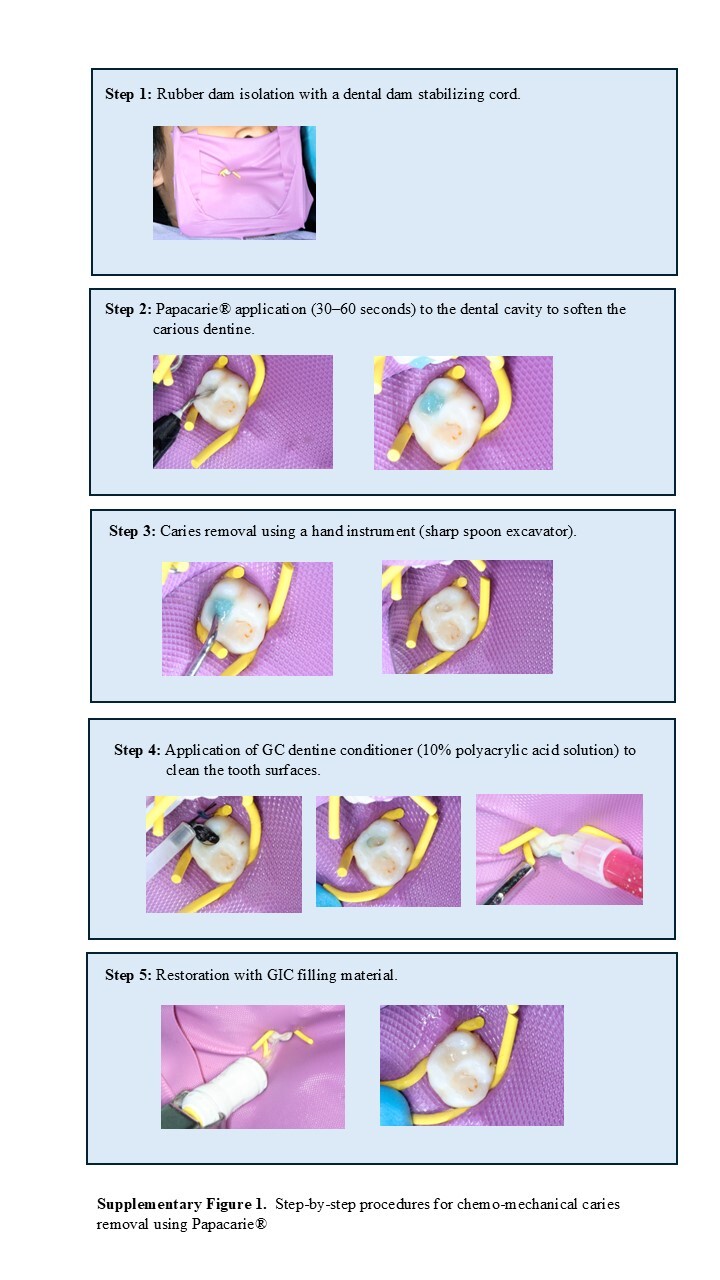

Supplement: Supplementary file 1 [file Image1.jpeg]

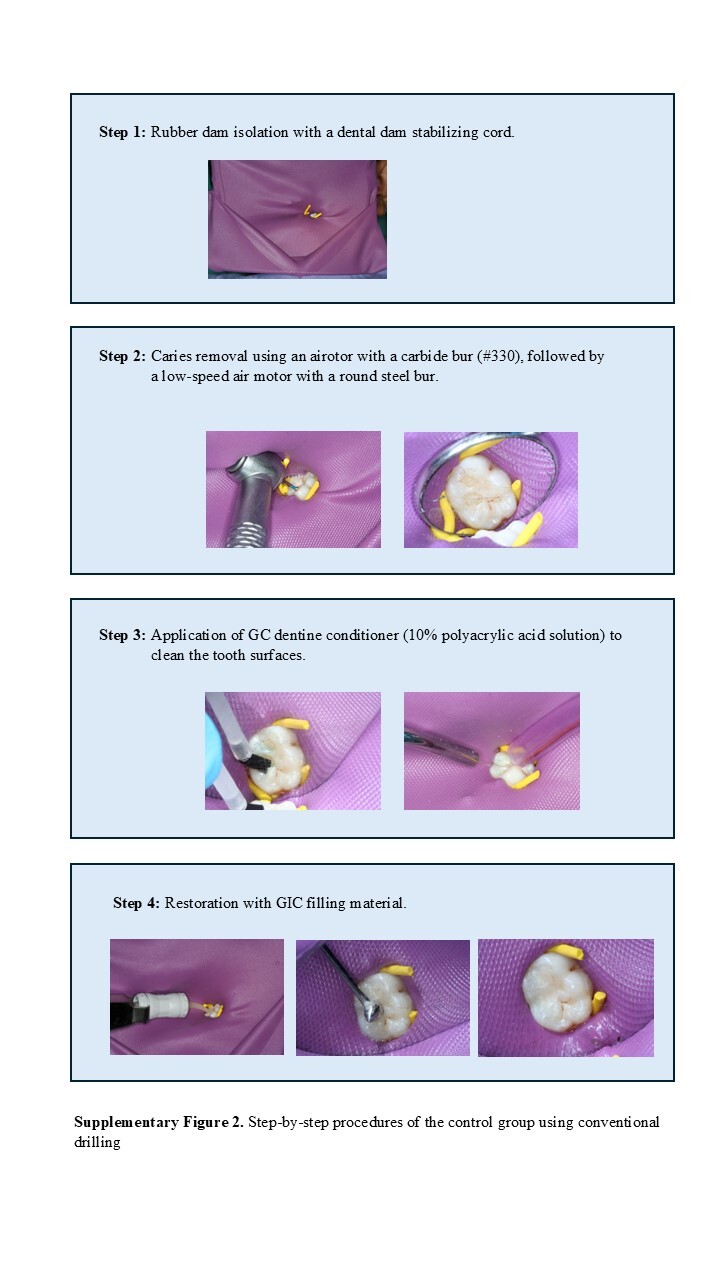

Supplement: Supplementary file 2 [file Image2.jpeg]
